# Integrated multi-omics profiling of amniotic fluid identifies predictive biomarkers for fetal growth restriction trajectories

**DOI:** 10.1080/07853890.2026.2718562

**Published:** 2026-08-15

**Authors:** Yan Cao, Wenjing Hao, Yan Wang, Chongchong Chai, Youran Li, Ying Liu, Hongyuan Zhu, Yifan Lu, Dong Wang, Yanhong Zhai, Yousheng Yan, Zheng Cao

**Affiliations:** ^a^ Department of Laboratory Medicine, Beijing Obstetrics and Gynecology Hospital, Beijing Maternal and Child Health Care Hospital, Capital Medical University, Beijing, China; ^b^ Center of Clinical Mass Spectrometry, Beijing Obstetrics and Gynecology Hospital, Beijing Maternal and Child Health Care Hospital, Capital Medical University, Beijing, China; ^c^ Department of Obstetrics, Beijing Obstetrics and Gynecology Hospital, Beijing Maternal and Child Health Care Hospital, Capital Medical University. Beijing, China; ^d^ Chinese Pharmacopoeia Commission, Beijing, China; ^e^ Shanghai AB Sciex Analytical Instrument Trading Co., Ltd, Shanghai, China; ^f^ Center for Prenatal Diagnosis, Beijing Obstetrics and Gynecology Hospital, Beijing Maternal and Child Health Care Hospital, Capital Medical University, Beijing, China

**Keywords:** Fetal growth restriction, amniotic fluid, Integrated multi-omics, prognostic stratification, biomarkers

## Abstract

**Background:**

Fetal growth restriction (FGR) is a complex condition with highly heterogeneous clinical outcomes, making prenatal distinction between transient and persistent growth failure challenging. This study aims to identify amniotic fluid (AF) biomarkers capable of differentiating distinct FGR trajectories and characterizing persistent growth failure mechanisms.

**Methods:**

Integrated proteomic and metabolomic profiling was performed on AF samples from transient FGR (*n* = 11), persistent FGR (*n* = 9), and healthy controls (*n* = 13). Diagnostic and prognostic models were developed using multivariate analysis. Selected protein candidates were validated *via* ELISA in an independent cohort (*n* = 69).

**Results:**

Multi-omics analysis revealed distinct molecular signatures for FGR stratification. A two-protein diagnostic panel (PDGFA and phospho-STAT5A) achieved an AUC of 1.000 in the discovery stage and 0.780 in the external validation cohort. For prognostic assessment, a molecular signature including IREB2, HLA-C, and PLXNB2 accurately predicted persistent growth failure from transient recovery (AUC = 0.966). Cross-platform integration highlighted the mass spectrometry-derived WASHC2C as a central hub protein with a significant progressive increase across the control, transient, and persistent groups (*p* < 0.001).

**Conclusions:**

This study establishes a multi-omics framework for prenatal FGR stratification. Our findings identify distinct molecular signatures reflecting the intrauterine environment and provide high-performance molecular tools for predicting divergent fetal growth trajectories to guide personalized clinical decision-making.

## 1. Introduction

Fetal growth restriction (FGR), defined as the failure of a fetus to reach its genetically predetermined growth potential, affects 8–10% of pregnancies and remains a primary driver of perinatal morbidity and mortality [1,2]. Typically diagnosed when the estimated fetal weight (EFW) falls below the 10th percentile, FGR is increasingly recognized as a precursor to maladaptive ‘metabolic programming’, elevating the risk of neurodevelopmental deficits and chronic metabolic diseases in later life [3,4]. Despite its clinical significance, current diagnostic strategies, ranging from symphysis-fundal height measurements with low sensitivity (approximately 16%) to operator-dependent ultrasonography, frequently struggle to achieve precise prognostic stratification [2].

Specifically, distinguishing between transient growth faltering and persistent pathological restriction remains a critical challenge. Clinically, fetuses presenting with identical growth restriction (EFW < 10^th^ percentile) in mid-gestation can harbor completely divergent developmental fates. While a subset of these cases undergoes progressive intrauterine deterioration that necessitates iatrogenic preterm delivery (persistent FGR), others successfully mount a third-trimester catch-up growth to achieve a normal birth weight at term (transient FGR). Currently, conventional ultrasound lacks the resolution to prospectively bifurcate these trajectories at the initial mid-trimester diagnosis [5]. This diagnostic gap underscores an urgent need for objective molecular biomarkers that transcend morphological assessment to enable early risk stratification and personalized prenatal management, ultimately improving the lifelong health trajectories of affected infants.

The molecular pathogenesis of FGR is inherently multifactorial, primarily stemming from placental insufficiency at the maternal-fetal interface. This is often driven by impaired spiral artery remodeling or inflammatory damage, which disrupts essential nutrient and oxygen exchange. Despite extensive research into oxidative stress and dysregulated inflammatory pathways, the precise cellular orchestrations underlying FGR remain incompletely elucidated [6,7]. Historically, single-omics studies utilizing maternal serum or umbilical cord blood have attempted to decode these processes; however, these specimens are prone to maternal metabolic interference and dilution, leading to inconsistent findings [8,9]. In contrast, AF serves as a superior liquid biopsy that provides a direct biological window into the intrauterine environment [10]. As a dynamic interface mirroring the functional integrity of the fetoplacental unit, AF offers a more authentic representation of fetal physiology than peripheral biofluids [2,11]. By integrating proteomics with metabolomics, a multi-omics approach provides a robust framework for resolving the holistic molecular landscape of FGR. This integration not only facilitates the elucidation of dysregulated molecular networks but also enables the identification of robust biomarker signatures essential for bridging the gap between conventional diagnosis and individualized prognostic assessment.

In this study, we established a well-characterized case-control study to perform integrated metabolomic and proteomic profiling of AF across three rigorously defined cohorts: appropriately grown fetuses (healthy controls), transient FGR, and persistent FGR. The primary objectives of this investigation were to [1]: comprehensively map the molecular landscape of AF in FGR [2]; identify key metabolites and proteins significantly linked to the persistence of growth restriction [3]; uncover dysregulated biological pathways through integrative bioinformatic analysis; and [4] construct a multi-omics biomarker model to predict neonatal prognosis. By dissecting the molecular disparities between these dynamic subgroups, this study seeks to elucidate the mechanisms underlying FGR progression versus recovery, thereby facilitating precise risk stratification and providing robust tools for personalized clinical management.

## 2. Methods

### 2.1. Study design and clinical sample collection

**Figure 1.**
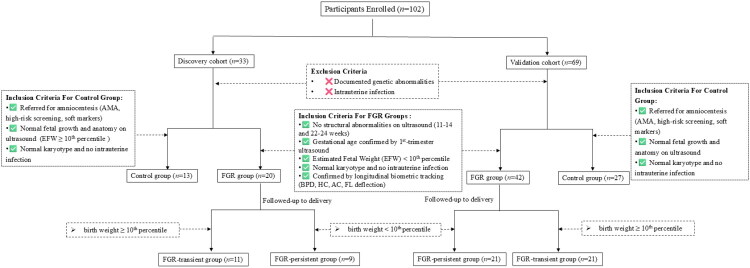
**Study design and workflow for the integrated proteometabolomic profiling of FGR**.

This study employed a two-stage case-control design, comprising a discovery cohort (*n* = 33) and an independent validation cohort (*n* = 69), enrolling 102 pregnant women undergoing clinical amniocentesis at Beijing Obstetrics and Gynecology Hospital between 2023 and 2024 (Figure 1). The study protocol was approved by the Ethics Committee of Beijing Obstetrics and Gynecology Hospital (Approval No. 2023-KY-009-01), with written informed consent obtained from all participants. To minimize clinical heterogeneity, FGR was defined by aligning prenatal ultrasound phenotypes with international Delphi consensus criteria to exclude constitutionally small-for-gestational-age (SGA) fetuses [12]. The control group (*n* = 40) comprised pregnancies with normal fetal growth trajectories (longitudinal EFW ≥10^th^ percentile), normal karyotypes, and no placental insufficiency or infection. The FGR group (*n* = 62), initially indicated by an EFW < 10^th^ percentile (first-trimester dating confirmed), was verified by documenting true fetal growth trajectory deflection or severe biometric asymmetry based on serial measurements of biparietal diameter (BPD), head circumference (HC), abdominal circumference (AC), and femur length (FL); chromosomal anomalies and congenital infections were strictly excluded. Based on longitudinal growth velocities and postnatal outcomes, the FGR cohort was stratified into [1]: transient FGR (Growth Recovery): fetuses with severe second/early-third trimester growth deceleration but demonstrating late-gestation catch-up growth, resulting in a birth weight ≥ 10^th^ percentile; and [2] Persistent FGR (Progressive Restriction): fetuses with unremitting prenatal growth failure, culminating in a birth weight consistently < 10^th^ percentile. Standardized AF samples were collected during amniocentesis and stored at −80 °C for proteometabolomic analysis.

### 2.2. Sample preparation

For metabolomics, samples were processed on an automated Starlid^™^ workstation *via* protein precipitation (1:1 methanol/acetonitrile) with isotope-labeled internal standards. Quality control (QC) samples were generated by pooling equal aliquots of all supernatants. For proteomics, proteins were extracted in SDS lysis buffer, quantified by BCA assay, and digested using the Filter-Aided Sample Preparation (FASP) protocol [13]. Resulting peptides were desalted prior to LC − MS/MS analysis. (Detailed procedures are provided in the Supplementary Materials.)

### 2.3. Untargeted metabolomics profiling analysis for AF

AF metabolomics was performed using a Vanquish UHPLC system coupled with a Q Exactive Mass Spectrometer (Thermo Fisher Scientific). Data were acquired in both ESI positive and negative ionization modes to maximize coverage. Metabolites were annotated by matching retention times, accurate mass, and MS/MS patterns against public databases. According to Metabolomics Standards Initiative (MSI) guidelines [14], 526 metabolites were identified at Level 1 and 1,347 at Level 2. (Detailed procedures are provided in the Supplementary Materials.)

### 2.4. Untargeted proteomics profiling analysis for AF

Proteomic profiling was conducted *via* LC − MS/MS in Data-Independent Acquisition (DIA) mode. Peptides were separated on an EASY-nLC 1200 system (Thermo Fisher Scientific) over a 60-minute gradient and analyzed using an Orbitrap Q Exactive HF mass spectrometer. DIA raw data were processed with Spectronaut 19 against the Human UniProt database, with the false discovery rate (FDR) strictly controlled at <1% at both peptide and protein levels. (Detailed procedures are provided in the Supplementary Materials.)

### 2.5. Enzyme-linked immunosorbent assay (ELISA)

AF levels of candidate biomarkers were validated using commercial ELISA systems. The concentrations of functional PDGF-AA homodimer (Catalog No. ELH-PDGFAA, RayBiotech, USA) and the activation levels of phosphorylated STAT5A at the Tyr694 site [phospho-STAT5A (Tyr694)] (Catalog No. PEL-STAT5A-Y694-T, RayBiotech, USA) were quantified *via* multi-step sandwich ELISA according to the manufacturer’s specific protocols. Additionally, human Plexin B2 (PLXNB2), Human Leukocyte Antigen C (HLA-C), and Iron-Responsive Element-Binding Protein 2 (IREB2) were determined using specific sandwich ELISA kits. All measurements were performed in duplicate, and data extraction was conducted *via* linear regression against respective standard curves. Detailed experimental procedures, incubation parameters, and assay sensitivities for each target are provided in the Supplementary Methods.

### 2.6. Statistical analysis

Statistical analyses were conducted using GraphPad Prism 8.0 and SIMCA-P 14.1. Data are expressed as mean ± SD. Normality was assessed *via* the Kolmogorov-Smirnov test. For normally distributed data, inter-group differences were analyzed using Student’s *t-test* or one-way ANOVA as appropriate. For non-normally distributed continuous variables, multi-group comparisons were evaluated using the Kruskal-Wallis test followed by Dunn’s post-hoc multiple comparisons test. Multivariate modeling, including principal component analysis (PCA), partial least squares-discriminant analysis (PLS-DA), and orthogonal PLS-DA (OPLS-DA), was performed to evaluate metabolic and proteomic profiles. Diagnostic accuracy was determined by Receiver Operating Characteristic (ROC) curve analysis, and biomolecular interaction networks were constructed using MetaboAnalyst 6.0. To ensure statistical adequacy, a post-hoc power calculation was performed for the independent validation cohort (*n* = 69). Given the large effect sizes observed for the selected multi-omics biomarkers (Cohen’s *d* > 0.8), a sample size of 69 provides a statistical power exceeding 85% (power > 0.85) at a two-sided significance level (*α*) of 0.05, demonstrating that the cohort was fully sufficient and adequately powered to validate the prognostic and diagnostic signatures.

## 3. Results

### 3.1. Clinical characteristics and outcomes of the study cohort

The maternal demographics, prenatal fetal sonographic parameters, and postnatal delivery outcomes of the study population (*n* = 102) are presented in Table 1. No statistically significant differences were observed among the healthy control (*n* = 40), transient FGR (*n* = 32), and persistent FGR (*n* = 30) groups regarding maternal age, pre-pregnancy body mass index (BMI), or gestational age at amniocentesis (*p* > 0.05). However, mothers in the persistent FGR group exhibited elevated systolic (125 ± 15 mmHg) and diastolic blood pressure (82 ± 11 mmHg) compared to controls (*p* < 0.05). Maternal biochemical profiling revealed that the transient FGR group already displayed baseline elevations in total bile acids (TBA: 8.9 ± 3.4 μmol/L), ALT (34.5 ± 12.8 U/L), and AST (29.8 ± 9.4 U/L) relative to controls (*p* < 0.05). These hepatobiliary alterations were further pronounced in the persistent FGR cohort (TBA: 14.6 ± 5.8 μmol/L; ALT: 85.2 ± 32.4 U/L; AST: 68.4 ± 24.1 U/L), which was also accompanied by a significant reduction in serum albumin (ALB: 34.2 ± 3.5 g/L, *p* < 0.05 *vs.* control).

**Table 1.** Clinical and demographic characteristics of the study population.

Feature	Control (*n* = 40)	Transient FGR (*n* = 32)	Persistent FGR (*n* = 30)	*p* Value (C *vs.* T *vs.* P)
Maternal Features				
Age (years)	32.5 ± 4.1	31.8 ± 3.8	33.1 ± 4.5	0.456
Pre-preg BMI (kg/m²)	21.4 ± 2.3	22.1 ± 2.8	21.8 ± 2.5	0.612
SBP (mmHg)	115 ± 10	118 ± 12	125 ± 15*	**0.045**
DBP (mmHg)	72 ± 8	75 ± 9	82 ± 11*	**0.038**
Maternal Biochemical Profiles				
Total Bile Acids (TBA, μmol/L)	4.2 ± 1.8	8.9 ± 3.4*	14.6 ± 5.8*^&^	**<0.001**
ALT (U/L)	18.4 ± 6.2	34.5 ± 12.8*	85.2 ± 32.4*^&^	**<0.001**
AST (U/L)	22.1 ± 5.5	29.8 ± 9.4*	68.4 ± 24.1*^&^	**<0.001**
ALB (g/L)	38.5 ± 3.2	36.4 ± 2.9	34.2 ± 3.5*	**0.012**
Fetal Sonographic Features (at Amniocentesis)				
Amniocentesis GA (wks)	24.2 ± 1.5	24.5 ± 1.2	23.8 ± 1.8	0.388
Biparietal Diameter (BPD, cm)	6.2 ± 0.5	6.0 ± 0.4	5.4 ± 0.6	**0.015**
Head Circumference (HC, cm)	22.4 ± 1.8	21.9 ± 1.5	20.1 ± 2.1*^&^	**0.008**
Abdominal Circumference (AC, cm)	20.5 ± 1.4	18.2 ± 1.2*	16.4 ± 1.9*^&^	**<0.001**
Femur Length (FL, cm)	4.5 ± 0.4	4.1 ± 0.3*	3.6 ± 0.5*^&^	**<0.001**
Amniotic Fluid Volume (AFV, cm)	5.4 ± 1.2	4.8 ± 1.1	3.8 ± 1.4*	**0.003**
Neonatal Delivery Outcomes				
Delivery GA (wks)	39.2 ± 1.1	38.5 ± 1.4*	36.4 ± 2.5*^&^	**<0.001**
Birth Weight (g)	3250 ± 310	2950 ± 280*	1850 ± 420*^&^	**<0.001**
Birth Weight Centile	>10^th^	>10^th^	<10^th^	**<0.001**
Neonatal Gender (Male/Female)	22 / 18	17 / 16	14 / 15	0.884

*Note:* Data are expressed as mean ± standard deviation or number. SBP, systolic blood pressure; DBP, diastolic blood pressure; ALT, alanine aminotransferase; AST, aspartate aminotransferase; ALB, albumin; TBA, total bile acids; GA, gestational age; BPD, biparietal diameter; HC, head circumference; AC, abdominal circumference; FL, femur length; AFV, amniotic fluid volume. Global *P*-values are calculated *via* one-way ANOVA, Kruskal-Wallis test, or Chi-square test as appropriate. Pairwise post-hoc comparisons were evaluated using Tukey’s HSD or Dunn’s multiple comparison test, *P* values in bold indicate statistical significance (*p* < 0.05). *p < 0.05 *vs.* Control group; ^&^*p* < 0.05 *vs.* Transient FGR group.

Prenatal sonographic biometrics captured during the amniocentesis window reflected distinct fetal growth trajectories. During sampling, the transient FGR group already demonstrated significant structural limitations compared to controls, evidenced by reductions in abdominal circumference (AC: 18.2 ± 1.2 cm *vs.* 20.5 ± 1.4 cm) and femur length (FL: 4.1 ± 0.3 cm *vs*. 4.5 ± 0.4 cm, both *p* < 0.05). This growth restriction was more severe in the persistent FGR cohort, which showed stepwise decreases in head circumference (HC: 20.1 ± 2.1 cm), AC (16.4 ± 1.9 cm), and FL (3.6 ± 0.5 cm) relative to both the control and transient groups (*p* < 0.05), alongside restricted amniotic fluid volumes (AFV: 3.8 ± 1.4 cm, *p* < 0.05 *vs*. control). This asymmetrical biometric pattern relative to HC is consistent with placental insufficiency-mediated growth restriction.

Postnatal endpoints further confirmed the divergent clinical courses of the stratified cohorts. The persistent FGR group required earlier delivery (36.4 ± 2.5 weeks) and presented with severely restricted birth weights (1850 ± 420 g, <10^th^ percentile, *p* < 0.05 *vs*. both groups). Conversely, the transient FGR group demonstrated late-gestation catch-up growth, achieving a significantly higher mean birth weight (2950 ± 280 g, >10^th^ percentile) and a longer gestational age at delivery (38.5 ± 1.4 weeks). These distinct clinical trajectories validate the phenotypic basis of our cohort stratification for subsequent proteometabolomic analysis.

### 3.2. Multi-omics landscape and diagnostic signatures of FGR

**Figure 2.**
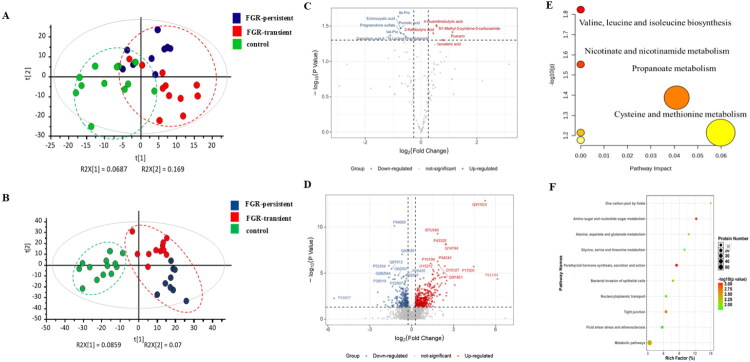
**Multi-omics profiling and functional enrichment analysis of amniotic fluid in FGR.** (A,B) Score plots of metabolites (A) and proteins (B) across the control and FGR groups. (C, D) Volcano plots of (C) DEMs and (D) DEPs associated with FGR. (E) Pathway enrichment analysis of DEMs based on the KEGG database. (F) Functional enrichment analysis of DEPs categorized by biological processes.

Integrated untargeted metabolomic and proteomic profiling identified 1,873 high-confidence metabolites and 4,132 proteins in AF samples (Figure S1). Tight clustering of quality control (QC) samples in unsupervised principal component analysis (PCA) confirmed high technical reproducibility (Figure S2). While metabolomic PCA showed overlapping profiles, proteomic PCA revealed a distinct separation trend between FGR and control groups. Supervised PLS-DA modeling further sharpened this segregation, particularly for the proteome, underscoring a robust molecular reprogramming in FGR (Figure 2A,B).

Differential expression analysis (VIP > 1.2, *p* < 0.05, |Fold Change| > 1.2) identified 8 metabolites (DEMs) and 188 proteins (DEPs; 127 upregulated, 61 downregulated) significantly altered in FGR (Figure 2C,D; Figure S3; Tables S1, S2). Unsupervised hierarchical clustering and quantitative bar plot analysis of these top-ranked differentially expressed molecules achieved clear group stratification and robustly demonstrated consistent molecular patterns across individual samples (Figure S4). Functional enrichment showed that DEMs were primarily involved in branched-chain amino acid biosynthesis (Valine, leucine, and isoleucine), nicotinate and nicotinamide metabolism, and cysteine and methionine metabolism (Figure 2E), while DEPs were enriched in the ‘One carbon pool by folate’ (Figure 2F). Joint pathway analysis (Figure S5) further integrated these findings, identifying purine metabolism and amino sugar and nucleotide sugar metabolism as the most significantly perturbed convergent pathways. This metabolic crosstalk linking one-carbon units to purine synthesis underscores a systemic impairment in nucleotide biosynthesis and redox homeostasis, thereby providing a systems-level view of the compromised intrauterine environment in persistent FGR.

### 3.3. Molecular signatures predicting FGR clinical prognosis

**Figure 3.**
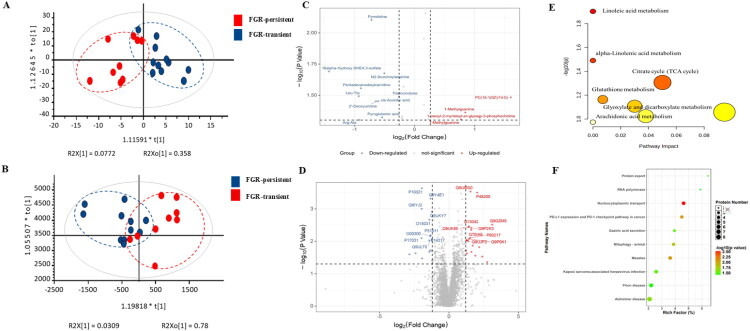
**Molecular signatures distinguishing transient FGR and persistent FGR.** (A,B) Score plots of metabolites (A) and proteins (B) across the transient FGR and persistent FGR groups. (C,D) Volcano plots of (C) DEMs and (D) DEPs associated with divergent growth trajectories. (E) Pathway enrichment analysis of DEMs based on the KEGG database. (F) Functional enrichment analysis of DEPs categorized by biological processes.

To identify molecular features predictive of clinical trajectories, we compared the transient and persistent FGR groups. Supervised OPLS-DA modeling demonstrated clear segregation between these two prognostic subgroups in both metabolomic and proteomic profiles (Figure 3A,B). Through differential expression analysis, we identified 14 DEMs and 65 DEPs significantly altered in the persistent group relative to the transient group (Figure 3C,D; Tables S3 and S4). Despite similar initial clinical presentations, these molecular signatures effectively discriminated divergent growth trajectories. The distinct clustering of these top-ranked differentially expressed molecules was further substantiated by unsupervised hierarchical clustering and quantitative bar plot analysis, which robustly demonstrated consistent individual expression patterns across the cohorts (Figure S6). Pathway enrichment analysis revealed that the metabolic perturbations specific to persistent FGR were predominantly centered on mitochondrial energy machinery, including the citrate cycle (TCA cycle) and glyoxylate metabolism, as well as pyrimidine metabolism (Figure 3E). In contrast, DEPs in the persistent group were primarily enriched in genetic information processing, including RNA polymerase activity, nucleocytoplasmic transport, and protein export (Figure 3F). Together, these multi-omics alterations map a distinct molecular profile for the persistent phenotype, highlighting coordinate changes across both energy metabolism pathways and protein expression machinery.

### 3.4. Integrated multi-omics and mechanistic model of FGR progression

**Figure 4.**
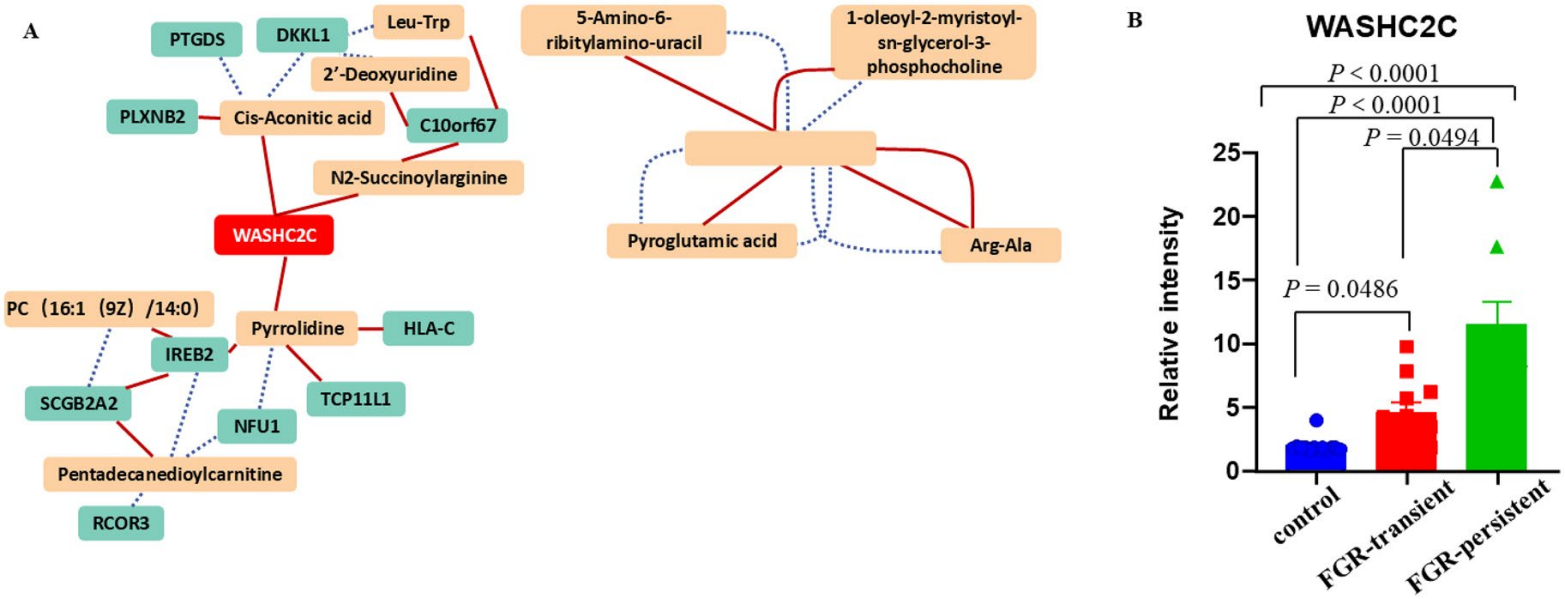
**Integration of multi-omics data and clinical correlation of WASHC2C.** (A) Correlation network integrating differential metabolites and potential protein targets in FGR. Nodes represent metabolites (green squares) and proteins (orange squares), with the central hub protein WASHC2C highlighted in red. Edges represent Spearman correlation coefficients (*r*); solid red lines indicate positive correlations (*r* > 0), while dashed blue lines indicate negative correlations (*r* < 0). Only significant interactions with *p* < 0.05 are displayed. (B) Relative abundance of WASHC2C derived from untargeted mass spectrometry proteomics in the control, transient FGR, and persistent FGR groups. (Global variance analyzed by Kruskal-Wallis test, *p* < 0.0001; pairwise comparisons evaluated *via* Dunn’s multiple comparisons test with exact adjusted *P*-values annotated on the graph).

To investigate the coordination between diagnostic and prognostic signatures, we performed cross-platform correlation and molecular interaction analyses (Figure S7). This integrative approach identified WASHC2C (Q9Y4E1), a core component of the WASH complex involved in endosomal trafficking, as a central hub node within the prognostic stratification network (Figure 4A). WASHC2C exhibited direct correlations with key trajectory-associated molecules, including the TCA cycle intermediate *cis*-aconitic acid, N2-succinoylarginine, and pyrrolidine. Concurrently, the multi-omics landscape also revealed a co-regulated metabolic cluster primarily composed of peptidic and nitrogenous metabolites, such as Arg-Ala and pyroglutamic acid, reflecting coordinated systemic metabolic shifts. Quantitatively, mass spectrometry-based proteomic profiling demonstrated a progressive increase in WASHC2C relative abundance across the clinical trajectories (Kruskal-Wallis test, *p* < 0.0001; Figure 4B). Post-hoc pairwise comparisons indicated that WASHC2C levels were already significantly elevated in the transient FGR group compared to healthy controls (*p* = 0.0486), and further escalated to peak levels in the persistent FGR cohort (*p* < 0.0001 *vs*. controls; *p* = 0.0494 *vs*. transient FGR).

**Figure 5.**
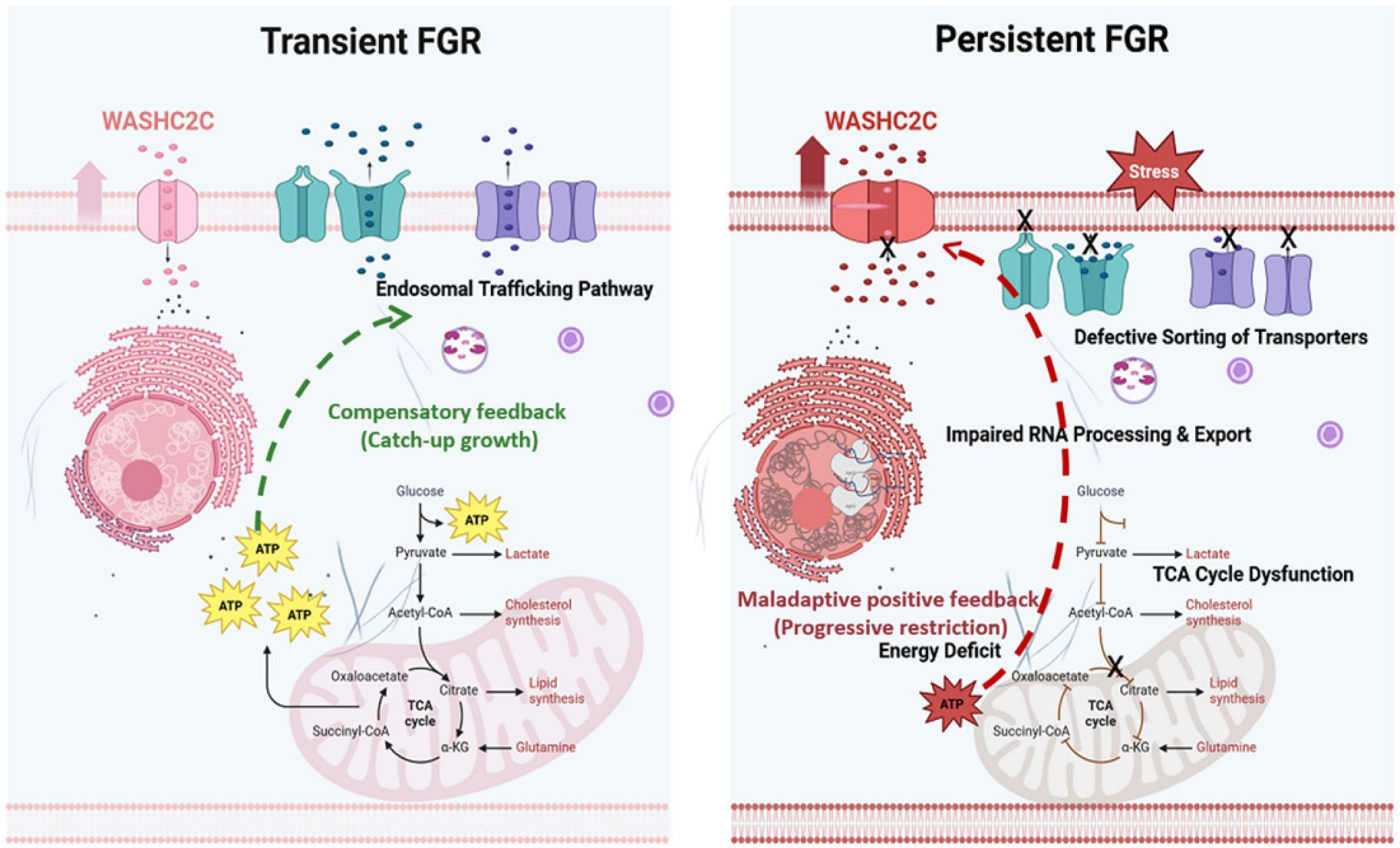
**Mechanistic model of WASHC2C-mediated growth failure trajectories in FGR.** The left panel depicts the transient trajectory, where initial restrictions are mitigated by compensatory pathways. The right panel outlines the persistent trajectory driven by severe WASHC2C overexpression, organelle distress, and metabolic crises. Dashed arrows indicate the respective adaptive and maladaptive feedback loops governing these distinct clinical outcomes.

Based on these molecular findings, we propose a mechanistic model for FGR progression and stratification (Figure 5). While transient FGR appears to involve limited alterations allowing for adaptive feedback mechanisms to facilitate homeostatic compensation and postnatal catch-up growth, persistent FGR is characterized by marked WASHC2C upregulation. We hypothesize that sustained WASHC2C overexpression may perturb the endosomal trafficking of critical nutrient transporters or growth factor receptors. This disruption of intracellular logistics compromises downstream nutrient signaling and exacerbates downstream metabolic crises, specifically mitochondrial dysfunction (TCA cycle impairment) and nucleotide synthesis deficiency. This systematic failure of metabolic and informational pathways establishes a maladaptive feedback cycle that locks the fetus into a persistent growth failure trajectory.

### 3.5. Validation of diagnostic and prognostic biomarkers

**Figure 6.**
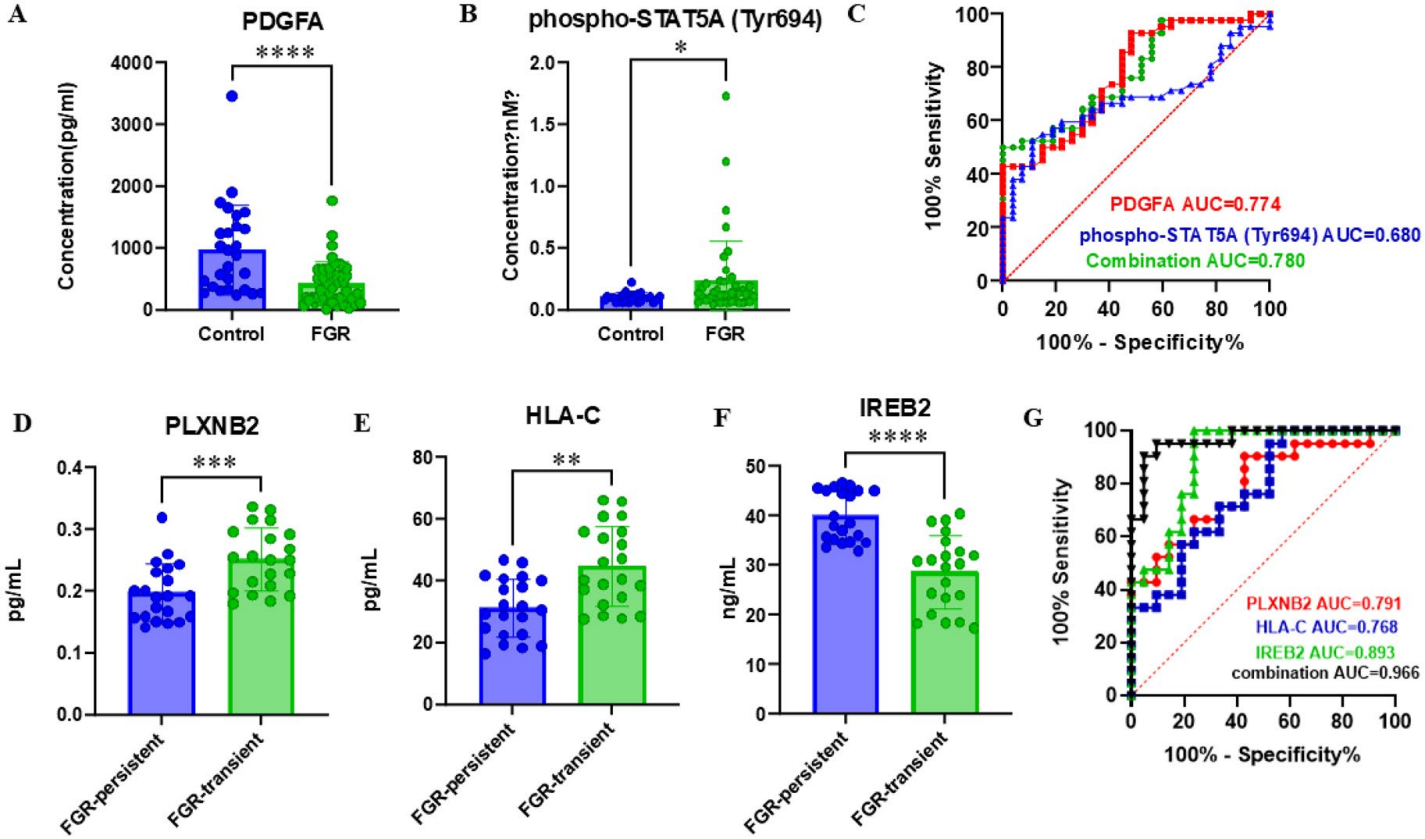
**External validation of diagnostic and prognostic biomarkers.** (A,B) Absolute concentrations of (A) PDGFA and (B) phospho-STAT5A (Tyr694) in the FGR and control groups. (C) ROC curve analysis of PDGFA, phospho-STAT5A (Tyr694), and their combined diagnostic model for FGR identification. (D–F) Absolute concentrations of (D) IREB2, (E) HLA-C, and (F) PLXNB2 across the transient and persistent FGR groups. (G) ROC curve analysis of IREB2, HLA-C, PLXNB2, and their combined model for prognostic stratification.

Within the discovery cohort, several proteins exhibited high discriminatory power between the FGR and control groups, with PDGFA and STAT5A achieving individual AUC values of 1.000 and 0.958, respectively (Table S2). A multivariate logistic regression model incorporating these two candidates maintained strong diagnostic capacity in the discovery set, yielding an AUC of 1.000 (95% CI: 1.000–1.000) (Figure S8A). Based on clinical translatability and immunoassay availability, this two-protein panel was prioritized for further validation. External validation in the independent cohort confirmed these findings, with AF concentrations of PDGFA being significantly lower in FGR pregnancies than in healthy controls (*p* < 0.0001, Figure 6A), whereas phospho-STAT5A (Tyr694) levels exhibited a significant elevation (*p* < 0.05, Figure 6B). In the validation cohort, PDGFA and phospho-STAT5A (Tyr694) yielded individual AUC values of 0.774 and 0.680, respectively (Figure 6C). Upon incorporating these markers into a multi-protein panel, the combined diagnostic model achieved a robust AUC of 0.780. While this represents an expected numerical attenuation from the initial discovery baseline, the multi-marker panel demonstrated sustained statistical significance in the independent population.

To further evaluate the prognostic utility of the trajectory-associated signatures, a panel consisting of IREB2, HLA-C, and PLXNB2 was assessed. In the discovery set, these three-protein panel already demonstrated high accuracy in discriminating persistent from transient FGR (Figure S8B). External validation in the independent cohort confirmed significant alterations in the absolute concentrations of these proteins across growth trajectories (Figure 6D–F). The combined prognostic model incorporating IREB2, HLA-C, and PLXNB2 achieved an AUC of 0.966 (Figure 6G).

## Discussion

FGR remains a significant challenge in perinatal medicine, primarily due to the difficulty in prenatally distinguishing between transient cases characterized by late-gestational weight acceleration and persistent cases suffering from progressive pathological restriction that requires preterm interventions. While neonates with transient FGR achieve quantitative postnatal catch-up growth and normal birth weight percentiles, recent evidence underscores that they are not entirely normal and often harbor substantial long-term metabolic programming and immediate neonatal risks comparable to persistent cases. As demonstrated by Ramos et al. [5] infants born at term after transient FGR do not exhibit an improved composite neonatal outcome compared to those with persistent FGR, showing elevated risks for specific complications such as neonatal hypoglycemia. This evidence indicates that early intrauterine restriction leaves a lasting molecular scar despite subsequent late-gestational weight acceleration, highlighting the critical clinical necessity of exploring prenatal molecular stratification rather than relying solely on static, late-stage birth weight outcomes. By integrating AF proteomics and metabolomics, this study identified distinct molecular signatures that provide robust diagnostic stratification and offer insights into the molecular landscape of FGR progression. Our findings demonstrate that the AF proteome offers superior discriminatory power over the metabolome in identifying FGR. Specifically, we validated a diagnostic panel comprising PDGFA and phospho-STAT5A (Tyr694) that exhibited high diagnostic performance. The downregulation of PDGFA and concomitant hyperphosphorylation of STAT5A (Tyr694) likely reflect a concerted impairment of placental angiogenesis and compensatory hormonal signaling [15,16]. This proteomic dysregulation appears to orchestrate a cascade of systemic metabolic alterations, characterized by disrupted amino acid and one-carbon metabolism. In particular, significant perturbations in serine and glycine metabolism likely compromise the methyl donor supply and redox balance [17]. This restricted biosynthetic environment not only constrains immediate fetal development but may also potentially underpin the long-term metabolic programming risks observed in FGR offspring [18], establishing a biochemical foundation for persistent growth failure. Notably, these metabolic shifts in AF, including upregulated branched-chain amino acid biosynthesis and disrupted sulfur amino acid pathways, are consistent with previously reported alterations in maternal serum and umbilical cord blood of FGR pregnancies [19–23]. Such alignment suggests that while the metabolic fingerprint of FGR is systemic, it is robustly detectable prenatally within the amniotic compartment, positioning amniotic fluid as a high-value medium for clinical stratification.

A key finding of this study is the identification of WASHC2C as a central hub node within the AF molecular network. The relative abundance of WASHC2C exhibited a progressive increase from healthy controls to transient FGR, reaching its peak in the persistent FGR cohort. This stepwise upregulation positions WASHC2C as a potential molecular indicator mirroring the clinical severity of intrauterine growth restriction, rather than a verified causal driver. While the WASH complex is established to regulate endosomal trafficking, its marked upregulation in persistent FGR may reflect an adaptive cellular response to a severely compromised nutrient environment [24]. Mechanistically, structural biochemistry has shown that specific segments of the WASHC2C subunit directly interface with the sorting nexin-retromer coat complex to drive membrane trafficking and cargo recycling from endosomes [25]. Consequently, the substantial accumulation of WASHC2C observed in our network implies a potential compensatory hyper-activation of this intracellular sorting machinery as cells optimize the recycling of scarce nutrients and signaling receptors under metabolic stress. Notably, functional studies have demonstrated that disrupting the interaction between the retromer core and the WASH complex leads to compromised endosomal protein sorting, which serves as a mechanistic driver in central neurodegenerative pathways [26]. Furthermore, genetic variations in the endosomal sorting network have been associated with altered neural development and neurotransmission in clinical models [27]. Given that WASHC2C-mediated endosomal dynamics are highly active during neurogenesis and blood-brain barrier maintenance, its altered profiles in the amniotic compartment during the second or early-third trimester may bear vital clinical implications. This altered molecular signature might serve as a prenatal proxy reflecting not only localized placental insufficiency but also early systemic stress that could influence fetal neurodevelopmental programming. Accordingly, this hub node represents a compelling candidate for advanced prenatal risk stratification.

The molecular characterization of the divergence between transient and persistent FGR further reveals that persistent cases are defined by a complex interplay of proteomic adaptation and metabolic collapse. Specifically, the enrichment of proteins involved in RNA processing, nucleocytoplasmic transport, and protein export suggests coordinated cellular responses to mitigate severe metabolic stress [28,29]. However, because these macromolecular synthesis and transport processes are inherently energy-dependent, their sustained activation likely exacerbates the existing energy deficit caused by the downregulation of the tricarboxylic acid (TCA) cycle and perturbed pyrimidine metabolism [30,31]. These findings align with established evidence of mitochondrial dysfunction and compromised ATP production in restriction-affected placentas [32–34]. Together, this molecular landscape suggests that persistent FGR is characterized by a maladaptive cycle of disrupted protein homeostasis and energy failure, locking the system into a progressive growth restriction phenotype.

In clinical biomarker development, transitioning from tightly controlled discovery cohorts to independent validation populations inherently entails performance attenuation [35]. Here, while our two-protein diagnostic panel experienced a typical validation shrinkage to an AUC of 0.780 in the independent cohort, this performance remains highly comparable to, or even outperforms, previously established perinatal signatures for gestational syndromes, which historically plateau between 0.70 and 0.80 [35,36]. Given the invasive nature and procedural risks of amniocentesis, a standalone screening tool with an AUC of 0.780 might appear clinically sub-optimal at first glance. However, these AF samples were clinical derivatives from patients already undergoing indicated procedures for standard routine rationales, such as advanced maternal age or abnormal serological screening. Consequently, utilizing this panel enables a valuable, risk-free opportunistic stratification within an already justified clinical workflow, rather than requiring an unindicated invasive intervention. In contrast to the diagnostic attenuation, our prognostic model combining IREB2, HLA-C, and PLXNB2 maintained high predictive performance with an AUC of 0.966. These biomarkers collectively capture the multi-dimensional nature of FGR pathophysiology by integrating iron homeostasis, maternal-fetal immune tolerance [37], and placental vascular remodeling. The capacity of this proteomic signature to accurately predict neonatal birth weight outcomes, even when initial ultrasound findings are comparable, demonstrates that the amniotic fluid proteome preserves critical prognostic memory of the gestational trajectory. These markers provide a multi-dimensional pathophysiological framework for the failure of catch-up growth, consistent with established roles of iron dysregulation and immune maladaptation in placental insufficiency [38,39]. While previous studies have primarily focused on static nutrient concentrations [7,8], our finding identifies the dynamic molecular signatures of the AF compartment as valuable tools for refined prenatal FGR management.

Several limitations of this study warrant consideration. First, although WASHC2C was prioritized as a central hub, its precise biological causality under nutrient restriction remains hypothetical due to the current lack of *in vitro* functional assays. Furthermore, its evaluation *via* traditional immunoassays was restricted by the unavailability of reliable commercial antibodies. To circumvent this technical barrier and facilitate clinical translation, the development of customized, antibody-independent parallel reaction monitoring (PRM) mass spectrometry workflows represents a necessary future avenue to reliably absolute-quantify this target. Second, our independent validation, while well-characterized, was evaluated within a single tertiary medical center without external multi-center validation. Additionally, detailed long-term pediatric neurodevelopmental outcomes and comprehensive neonatal complication profiles were not systematically tracked within this discovery-phase study, though prenatal cranial sonographic metrics (BPD/HC) and hard birth outcomes were fully captured. Lastly, the reliance on AF limits its capacity for universal clinical screening. Future prospective investigations are warranted to cross-validate these signatures within maternal plasma exosomes or placental extracellular vesicles, potentially facilitating a transition toward non-invasive, blood-based prenatal monitoring.

## Conclusion

In summary, this study demonstrates that integrated amniotic fluid proteometabolomic profiling can effectively differentiate between transient and persistent FGR. We established robust diagnostic and prognostic models to stratify FGR and predict divergent fetal growth trajectories, providing high-value tools for prenatal management. Our findings further characterize the systemic alterations associated with persistent growth failure, highlighting WASHC2C as a potential molecular indicator mirroring disease severity. These results offer a valuable framework to support precision clinical decision-making and provide a foundation for future research into the biological pathways underlying FGR progression. Ultimately, future translational efforts should investigate whether these amniotic fluid-derived signatures can be cross-validated in maternal circulation, thereby facilitating the development of a non-invasive stratification paradigm for widespread clinical implementation.

## Supplementary Material

Supplemental_materials.docx

## Data Availability

All processed multi-omics data supporting the findings of this study are provided within the article and its Supplementary Materials. Due to ethical regulations and privacy protection regarding human clinical specimens, the raw mass spectrometry datasets and individual patient clinical information are not publicly deposited. However, these de-identified raw datasets are available from the corresponding author (Prof. Zheng Cao) upon reasonable academic request.
