# Supplementary material for: Integrated multi-omics profiling of amniotic fluid identifies predictive biomarkers for fetal growth restriction trajectories
Source: Ann Med. 2026 Aug 15;58(1):2718562. doi: 10.1080/07853890.2026.2718562 (PMC13479514; doi:10.1080/07853890.2026.2718562)
Supplement: Supplementary file 1 [file IANN_A_2718562_SM7483.docx]

**Supporting Information**

**Integrated multi-omics profiling of amniotic fluid identifies predictive biomarkers for fetal growth restriction trajectories**

Yan Cao ^1,2†^, Wenjing Hao ^4†^, Yan Wang^1,2†^, Chongchong Chai^5^, Youran Li^1,2^, Ying Liu^1,2^, Hongyuan Zhu^1,2^, Yifan Lu ^1,2^, Dong Wang^6^, Yanhong Zhai^1,2^, Yousheng Yan^3*^, Zheng Cao^1,2*^

^1^Department of Laboratory Medicine, Beijing Obstetrics and Gynecology Hospital, Capital Medical University. Beijing Maternal and Child Health Care Hospital, Beijing, China

^2^Center of Clinical Mass Spectrometry, Beijing Obstetrics and Gynecology Hospital, Capital Medical University. Beijing Maternal and Child Health Care Hospital, Beijing, China

^3^Center for Prenatal Diagnosis, Beijing Obstetrics and Gynecology Hospital, Capital Medical University. Beijing Maternal and Child Health Care Hospital, Beijing, China

^4^Department of Obstetrics, Beijing Obstetrics and Gynecology Hospital, Capital Medical University. Beijing Maternal and Child Health Care Hospital, Beijing, China

^5^Chinese Pharmacopoeia Commission, Beijing, China

^6^Shanghai AB Sciex Analytical Instrument Trading Co., Ltd., Shanghai, China

^†^Yan Cao, Wenjing Hao and Yan Wang contributed equally to this work and share first authorship.

^*^ Corresponding author

**Zheng Cao**

Beijing Obstetrics and Gynecology Hospital, Capital Medical University

251 Yaojiayuan Road, Beijing, China 100026.

Tel: 86-10-52276406

Email: [zhengcao2011@ccmu.edu.cn](mailto:zhengcao2011@ccmu.edu.cn) and zhengcao2011@hotmail.com

**Yousheng Yan**

Beijing Obstetrics and Gynecology Hospital, Capital Medical University

251 Yaojiayuan Road, Beijing, China 100026.

Tel: 86-10-52276406

Email: yys_521@ccmu.edu.cn

**Supplementary Methods**

**Sample Collection and Storage**

Amniotic fluid samples were centrifuged at 3000 × *g* for 10 min at 4°C immediately after collection to remove cellular debris. Supernatants were aliquoted into cryovials and stored at −80 °C until further processing.

**Metabolomic Sample Preparation**

Frozen samples were thawed on ice prior to extraction. Using an automated Starlid™ workstation, 100 μL of each sample was transferred to a 96-well protein precipitation plate. Then, 400 μL of extraction solution (methanol:acetonitrile = 1:1, v/v) containing a mix of isotope-labeled internal standards was added. The mixture was vortexed at 750 rpm for 5 min and incubated for another 5 min at room temperature. After filtration through a 0.22 μm membrane, the supernatant was collected for LC−MS analysis. Quality control (QC) samples were generated by combining equal volumes of supernatant from all individual samples and were injected every 10 samples during the run to monitor instrument stability.

**Proteomic Sample** **Preparation**

Samples were lysed in buffer containing 4% SDS, 100 mM Tris/HCl (pH 7.6), and 0.1 M DTT at 95°C for 10 min. Protein concentration was determined using the BCA assay (Pierce™). An aliquot containing 50 μg of protein from each sample was processed using the FASP method(1). Briefly, proteins were reduced, alkylated, and digested with trypsin overnight at 37°C. Peptides were desalted using C18 StageTips, dried completely in a Speed-Vac concentrator, and reconstituted in 0.1% formic acid for LC-MS/MS analysis.

**Untargeted metabolomics profiling analysis for amniotic fluid**

Untargeted metabolomic profiling of amniotic fluid samples was performed using an ultra-high-performance liquid chromatography (UHPLC) system (Thermo Fisher Scientific, USA). Two microliters of the sample were injected into an Acquity BEH Amide column (1.7 μm, 2.1 mm × 100 mm, Waters, Milford, MA, United States) under a flow rate of 0.3 mL/min. The mobile phase consisted of solvent A (aqueous phase containing 25 mM ammonium acetate and 25 mM ammonium hydroxide) and solvent B (acetonitrile). The gradient elution was performed as follows: 0–0.5 min, 95% B; 0.5–7 min, 95–65% B; 7–8 min, 65–40% B; 8–9 min, 40–95% B; 9–11.9 min, 95% B. The mass spectrometric detection of metabolites was performed on Orbitrap Exploris 120 (Thermo Fisher Scientific, Waltham, MA, United States) with an ESI ion source. Data were acquired in both full-scan MS and data-dependent MS/MS modes. The key instrument parameters were set as follows: sheath gas flow rate, 50 Arb; auxiliary gas flow rate, 15 Arb; capillary temperature, 320 °C; full MS resolution, 60,000; MS/MS resolution, 15,000; stepped collision energy (SNCE), 20, 30, and 40 eV; and spray voltage, 3.8 kV in positive ion mode and –3.4 kV in negative ion mode.

**Untargeted proteomics profiling analysis for amniotic fluid**

Untargeted proteomic analysis of amniotic fluid samples was conducted using liquid chromatography-tandem mass spectrometry (LC-MS/MS) operated in data-independent acquisition (DIA) mode. Chromatographic separation was carried out on a homemade analytical column (75 μm i.d. × 20 cm, packed with 1.9 μm particles) with mobile phase A (0.1% formic acid in water) and mobile phase B (80% acetonitrile with 0.1% formic acid). A 60-minute gradient was applied as follows: 6% to 30% B (0–42 min), 30% to 42% B (42–51 min), 42% to 95% B (51–56 min), followed by a 4-minute hold at 95% B, with a constant flow rate of 300 nL/min. Mass spectrometry was performed using a Nanospray Flex ion source, with the spray voltage set to 2.3 kV and the ion transfer tube maintained at 320 °C. Full MS scans were acquired at a resolution of 120,000 across the *m*/*z* range of 398–900, using an AGC target of 3e^6^ and a maximum injection time of 80 ms. MS/MS spectra were collected in DIA mode with 32 variable windows at a resolution of 30,000, an AGC target of 3e^6^, a collision energy of 28%, and an automatic maximum injection time. Data processing was performed using Spectronaut 19, with trypsin/P specified as the protease allowing up to two missed cleavages. All other parameters were set to default(2).

**Detailed Protocols for RayBiotech ELISA Assays**

The quantifications of functional platelet-derived growth factor AA (PDGF-AA) homodimers and phosphorylated STAT5A (Tyr694) in amniotic fluid were conducted using commercial multi-step sandwich ELISA systems (RayBiotech, USA). For the PDGF-AA assay, standards and clinical samples were incubated in 96-well microplates pre-coated with an anti-human PDGF-AA capture antibody at room temperature under gentle shaking, followed by sequential incubations with a biotinylated anti-human PDGF-AA detection antibody and horseradish peroxidase (HRP)-conjugated streptavidin after thorough washing cycles. Concurrently, for the phospho-STAT5A (Tyr694) assay, amniotic fluid samples were initially pretreated with a dedicated cell lysate buffer containing protease and phosphatase inhibitors to stabilize the target, and then transferred to microplates pre-coated with an anti-pan-STAT5A antibody for target immobilization; subsequent target detection was achieved *via* consecutive incubations with a rabbit anti-phospho-STAT5A (Tyr694) antibody and an HRP-conjugated anti-rabbit IgG secondary antibody. Both assay pathways concluded with a unified colorimetric development phase, where TMB One-Step Substrate Reagent was added to individual wells for enzymatic amplification, incubated in the dark, and subsequently arrested using a sulfuric acid stop solution (0.2M H_2_SO_4_) prior to absorbance measurement at a wavelength of 450 nm. All experimental standards, controls, and patient specimens were systematically processed in duplicate to ensure data fidelity.

**Detailed Protocols for One-Step Sandwich ELISA Assays**

Quantifications of human Plexin B2 (PLXNB2), Human Leukocyte Antigen C (HLA-C), and Iron-Responsive Element-Binding Protein 2 (IREB2) in amniotic fluid were executed utilizing a rapid, single-stage sandwich ELISA framework. Briefly, 10 μL of amniotic fluid sample was introduced into target-specific 96-well microplates pre-coated with respective capture antibodies, immediately followed by the sequential addition of 40 μL of sample diluent and 100 μL of horseradish peroxidase (HRP)-conjugated detection antibody to establish a simultaneous reaction mixture within each well. This unified reaction assembly was subsequently incubated at 37°C for 60 minutes. Following incubation, the microplates were subjected to five consecutive washing cycles with wash buffer to eliminate residual matrices and unbound components. The colorimetric signal generation was then visualized by introducing TMB substrate and incubating the plates at 37°C for 15 minutes in the dark, which was subsequently arrested using a dedicated stop solution. The final absorbance values were extracted by reading the optical density (OD) at a wavelength of 450 nm. Absolute concentrations were determined *via* linear regression analysis against generated standard curves, with mathematical adjustments integrating a 5-fold dilution factor where applicable. The established analytical limits of detection for these assays were <1.0 pg/mL for both PLXNB2 and HLA-C, and <1.0 ng/mL for IREB2, with all experimental standards, quality controls, and clinical specimens systematically processed in duplicate to ensure data fidelity.

**References**

1.	Wisniewski JR, Zougman A, Nagaraj N, Mann M. Universal sample preparation method for proteome analysis. Nat Methods. 2009;6(5):359-62.

2.	Martinez-Val A, Bekker-Jensen DB, Hogrebe A, Olsen JV. Data Processing and Analysis for DIA-Based Phosphoproteomics Using Spectronaut. Methods Mol Biol. 2021;2361:95-107.

**Supplementary Figure S1 Overview of identified metabolites and proteins. (A)** Bar plot showing the class distribution of all identified metabolites. **(B)** Histogram showing the molecular weight (kDa) distribution of all identified proteins.

**Supplementary Figure S2 Principal component analysis (PCA) of metabolomic and proteomic profiles showing data quality and group distribution.** **(A)** PCA score plot derived from untargeted metabolomics data of amniotic fluid samples. **(B)** PCA score plot derived from untargeted proteomics data of the same sample set.

**Supplementary Figure S3 OPLS-DA models and validation tests for discriminating FGR from controls based on metabolomic and proteomic profiles. (A)** Score plot of the OPLS-DA model derived from metabolomic data comparing the FGR group versus the control group. **(B)** Permutation test (100 permutations) for the metabolomics OPLS-DA model. (C) Score plot of the OPLS-DA model derived from proteomic data for the same group comparison. (D) Permutation test (100 permutations) for the proteomics OPLS-DA model.

**Supplementary Figure S4. Unsupervised hierarchical clustering and quantitative validation of differentially expressed molecules between the control and FGR groups. (A)** Heatmap showing the relative abundance of significantly altered metabolites across individual samples from the control and FGR groups. **(B)** Quantitative bar plots with explicit error bars representing the relative expression levels of the top 5 most significantly altered metabolites sorted by *P*-values. **(C)** Heatmap showing the relative expression levels of significantly altered proteins across the same sample sets. **(D)** Quantitative bar plots with explicit error bars representing the relative expression levels of the top 10 most significantly altered proteins sorted by *P*-values.

**Supplementary Figure S5** Joint pathway analysis integrating proteomic and metabolomic profiles in FGR.

**Supplementary Figure S6 Permutation tests validating the OPLS-DA models for discriminating FGR-transient from FGR-persistent.** **(A)** Permutation test (100 permutations) for the OPLS-DA model derived from metabolomic data comparing FGR-transient versus FGR-persistent groups. **(B)** Permutation test (100 permutations) for the OPLS-DA model derived from proteomic data for the same group comparison.

**Supplementary Figure S6. Unsupervised hierarchical clustering and quantitative validation of differentially expressed molecules between the FGR-transient and FGR-persistent groups.** **(A)** Heatmap showing the relative abundance of significantly altered metabolites across individual samples from the FGR-transient and FGR-persistent groups. **(B)** Quantitative bar plots with explicit error bars representing the relative expression levels of the top 5 most significantly altered metabolites sorted by *P*-values. **(C)** Heatmap showing the relative expression levels of significantly altered proteins across the same sample sets. **(D)** Quantitative bar plots with explicit error bars representing the relative expression levels of the top 10 most significantly altered proteins sorted by *P*-values.

**Supplementary Figure S7. Integration of multi-omics signatures *via* cross-platform correlation analysis. (A)** Correlation heatmap of diagnostic molecular signatures. This matrix illustrates the pairwise Spearman correlation coefficients between DEMs and DEPs identified in the FGR *vs.* Control groups. The scale bar represents the correlation coefficient (*r*), where red indicates a positive correlation and blue indicates a negative correlation. The size and color intensity of the circles are proportional to the strength of the correlation. **(B)** Correlation heatmap of prognostic molecular signatures. Integrative analysis of DEMs and DEPs associated with growth trajectories (Transient *vs.* Persistent FGR).

**Supplementary Figure S8 Diagnostic and prognostic performance of candidate protein markers in the discovery cohort. (A)** ROC curve analysis for FGR diagnosis (FGR *vs.* Control). **(B)** ROC curve analysis for prognostic stratification (Transient *vs.* Persistent FGR).

**Table S1** Differentially expressed metabolites between the control and FGR groups.

| Identity | FC(FGR/Control) | *P* value | VIP | AUC |
| --- | --- | --- | --- | --- |
| Ile-Pro | 0.569916 | 0.0231 | 1.73403 | 0.7115 |
| 4-Guanidinobutyric acid | 1.466252 | 0.0307 | 1.43639 | 0.7481 |
| N1-Methyl-2-pyridone-5-carboxamide | 1.472859 | 0.032 | 1.89929 | 0.7308 |
| 2-Ketobutyric acid | 1.354692 | 0.04 | 1.67213 | 0.7269 |
| Pregnenolone sulfate | 0.605783 | 0.04 | 1.61013 | 0.7000 |
| Val-Pro | 0.651506 | 0.0414 | 1.38555 | 0.6731 |
| N-Lactoyl-Phenylalanine | 0.680266 | 0.0443 | 1.68544 | 0.6192 |
| Isovaleric acid/ Valeric acid | 1.718674 | 0.0499 | 1.57705 | 0.6692 |

**Table S2** Differentially expressed proteins between the control and FGR groups.

| PG.Protein  Accessions | PG.Genes | FC(FGR/Con) | *P* value | VIP | AUC |
| --- | --- | --- | --- | --- | --- |
| Q9Y5G3 | PCDHGB1 | 38.09994 | 1.3E-13 | 2.52811 | 0.9500 |
| P61244 | MAX | 5.654982 | 6.43E-05 | 1.75702 | 0.7923 |
| Q14194 | CRMP1 | 5.463141 | 7.18E-09 | 2.14661 | 0.9308 |
| P43320 | CRYBB2 | 5.371493 | 7.12E-09 | 1.95156 | 0.9308 |
| Q5SW96 | LDLRAP1 | 5.343973 | 2.75E-05 | 1.65344 | 0.8885 |
| O15127 | SCAMP2 | 5.132366 | 1.72E-06 | 1.93531 | 0.8923 |
| Q8N201 | INTS1 | 5.062818 | 2.58E-05 | 1.69976 | 0.9077 |
| Q8IYJ3 | SYTL1 | 4.821393 | 1.02E-05 | 1.90378 | 0.9154 |
| P42229 | STAT5A | 4.477453 | 2.69E-05 | 2.30302 | 0.9577 |
| O43424 | GRID2 | 4.141455 | 0.000197 | 1.74705 | 0.8577 |
| O75618 | DEDD | 4.07356 | 0.002175 | 1.75982 | 0.8115 |
| A0A087WSZ0 | IGKV1D-8 | 4.044153 | 3.14E-05 | 1.661 | 0.8231 |
| Q02078 | MEF2A | 3.953238 | 0.002159 | 1.77615 | 0.8000 |
| P46736 | BRCC3 | 3.868439 | 0.000296 | 1.58091 | 0.7808 |
| O95684 | CEP43 | 3.830758 | 1.62E-05 | 1.59746 | 0.8346 |
| Q9Y4E1 | WASHC2C | 3.810699 | 1.3E-06 | 1.86657 | 0.9269 |
| Q02224 | CENPE | 3.788717 | 4.41E-06 | 2.22142 | 0.9115 |
| B7U540 | KCNJ18 | 3.62994 | 5.05E-10 | 2.27824 | 0.9654 |
| Q8WV28 | BLNK | 3.620153 | 1.92E-05 | 2.05723 | 0.8462 |
| P84243 | H3-3A | 3.615681 | 5.36E-07 | 2.45578 | 0.9077 |
| O95292 | VAPB | 3.579517 | 0.000144 | 2.21805 | 0.8731 |
| P84095 | RHOG | 3.542662 | 9.34E-06 | 1.7723 | 0.9000 |
| P32320 | CDA | 3.40061 | 0.000649 | 1.54433 | 0.7692 |
| Q8IXQ4 | GPALPP1 | 3.242671 | 3.81E-05 | 1.80522 | 0.8077 |
| P36954 | POLR2I | 3.241577 | 0.001291 | 1.82435 | 0.8038 |
| P23109 | AMPD1 | 3.13234 | 0.000663 | 1.78785 | 0.8115 |
| P50479 | PDLIM4 | 3.103608 | 0.001287 | 1.61664 | 0.7692 |
| Q8TF01 | PNISR | 3.061887 | 0.002647 | 1.74639 | 0.8308 |
| Q8WYL5 | SSH1 | 3.003272 | 0.000321 | 1.76765 | 0.9077 |
| Q8WW12 | PCNP | 2.99502 | 3.4E-05 | 2.19515 | 0.8962 |
| Q3KQV9 | UAP1L1 | 2.979267 | 6.31E-06 | 2.27526 | 0.9115 |
| Q9NWZ3 | IRAK4 | 2.89081 | 0.002466 | 1.77176 | 0.8269 |
| P62760 | VSNL1 | 2.884476 | 0.001756 | 1.87561 | 0.8154 |
| P52594 | AGFG1 | 2.839904 | 1.33E-05 | 1.87278 | 0.8692 |
| O43598 | DNPH1 | 2.790292 | 1.39E-05 | 1.81329 | 0.9038 |
| Q9ULE0 | WWC3 | 2.741333 | 0.000408 | 1.62085 | 0.7885 |
| Q9H8H3 | TMT1A | 2.699485 | 0.003026 | 1.50655 | 0.7846 |
| O43252 | PAPSS1 | 2.628279 | 7.63E-05 | 1.95606 | 0.8231 |
| Q9Y2D5 | PALM2AKAP2 | 2.621146 | 0.00131 | 1.85424 | 0.7962 |
| P42677 | RPS27 | 2.619292 | 0.00044 | 1.96441 | 0.8846 |
| P16104 | H2AX | 2.560193 | 8.78E-07 | 2.27905 | 0.9192 |
| O95817 | BAG3 | 2.535671 | 0.003083 | 2.07372 | 0.8731 |
| Q8NI35 | PATJ | 2.528039 | 0.000244 | 2.00276 | 0.8654 |
| P22694 | PRKACB | 2.520515 | 0.000154 | 1.93026 | 0.8615 |
| Q7L8J4 | SH3BP5L | 2.490912 | 0.000191 | 2.029 | 0.8885 |
| Q14512 | FGFBP1 | 2.47865 | 0.000902 | 1.64382 | 0.7962 |
| Q13683 | ITGA7 | 2.451176 | 0.000891 | 1.55389 | 0.8269 |
| P01597 | IGKV1-39 | 2.417541 | 0.000164 | 1.83629 | 0.8769 |
| Q9Y5U2 | TSSC4 | 2.414504 | 0.018907 | 1.54498 | 0.7615 |
| P13984 | GTF2F2 | 2.401362 | 0.004994 | 1.90287 | 0.8231 |
| Q6QNY0 | BLOC1S3 | 2.398851 | 0.000316 | 2.21409 | 0.9192 |
| Q9NRL3 | STRN4 | 2.325405 | 0.000116 | 1.87324 | 0.7615 |
| O00193 | SMAP | 2.298748 | 0.002424 | 1.51266 | 0.8000 |
| P51159 | RAB27A | 2.277121 | 0.001057 | 1.82096 | 0.8000 |
| Q9BY76 | ANGPTL4 | 2.268172 | 0.001456 | 1.64688 | 0.7731 |
| Q15075 | EEA1 | 2.248231 | 0.001396 | 2.03192 | 0.8538 |
| Q86UY5 | FAM83A | 2.234626 | 0.006782 | 1.56575 | 0.7538 |
| Q6GYQ0 | RALGAPA1 | 2.234158 | 0.000237 | 1.84012 | 0.8192 |
| Q17RF5 | ODAPH | 2.228302 | 0.001014 | 1.67334 | 0.8192 |
| P43234 | CTSO | 2.179487 | 0.00017 | 2.28771 | 0.9231 |
| Q6ZU69 | SPATA31F1 | 2.161393 | 0.002561 | 1.59001 | 0.7769 |
| P41227 | NAA10 | 2.155606 | 0.000683 | 1.90718 | 0.9000 |
| P78347 | GTF2I | 2.135441 | 0.015218 | 1.81183 | 0.8077 |
| O00330 | PDHX | 2.073027 | 0.004166 | 1.80123 | 0.8077 |
| Q53TS8 | C2CD6 | 2.030915 | 0.001451 | 1.99883 | 0.8231 |
| Q99615 | DNAJC7 | 2.030374 | 0.000656 | 1.86842 | 0.8615 |
| Q15599 | NHERF2 | 2.024777 | 0.020749 | 1.56949 | 0.7615 |
| P61513 | RPL37A | 2.022101 | 0.005019 | 1.66606 | 0.7731 |
| Q8IUG5 | MYO18B | 2.021757 | 0.004217 | 1.59683 | 0.7654 |
| P62899 | RPL31 | 1.993297 | 0.005849 | 1.97374 | 0.8808 |
| Q96K83 | ZNF521 | 1.984775 | 0.000777 | 1.78587 | 0.8038 |
| Q6WCQ1 | MPRIP | 1.983723 | 0.008871 | 1.61288 | 0.7654 |
| P17535 | JUND | 1.979972 | 0.007247 | 1.50285 | 0.7692 |
| Q96DC8 | ECHDC3 | 1.979829 | 0.001633 | 1.77599 | 0.8192 |
| Q9BY84 | DUSP16 | 1.977844 | 0.000161 | 1.9152 | 0.8615 |
| Q8N8V2 | GBP7 | 1.971422 | 0.007913 | 1.74958 | 0.7731 |
| P52789 | HK2 | 1.961205 | 0.01098 | 1.63059 | 0.7462 |
| P42768 | WAS | 1.958508 | 0.005915 | 1.62426 | 0.7692 |
| O95359 | TACC2 | 1.949649 | 0.0032 | 1.76948 | 0.8231 |
| P48594 | SERPINB4 | 1.946821 | 0.005031 | 1.53769 | 0.7923 |
| O75915 | ARL6IP5 | 1.936288 | 0.004525 | 1.66186 | 0.7885 |
| Q3B7J2 | GFOD2 | 1.930877 | 0.000189 | 1.84075 | 0.7962 |
| Q8ND56 | LSM14A | 1.927954 | 0.000672 | 1.74576 | 0.9038 |
| Q8NBN7 | RDH13 | 1.924889 | 0.004519 | 1.58812 | 0.7923 |
| P62328 | TMSB4X | 1.923549 | 0.000681 | 1.66952 | 0.8654 |
| O75054 | IGSF3 | 1.909933 | 0.027822 | 1.61884 | 0.7692 |
| Q6P4E1 | GOLM2 | 1.900005 | 0.001963 | 1.90169 | 0.8346 |
| Q9ULU4 | ZMYND8 | 1.885143 | 0.026086 | 1.56803 | 0.7577 |
| Q86TY3 | ARMH4 | 1.884647 | 0.01421 | 1.50275 | 0.7269 |
| Q01469 | FABP5 | 1.870888 | 0.00456 | 1.59572 | 0.7846 |
| Q12872 | SFSWAP | 1.856254 | 0.00055 | 2.13449 | 0.9038 |
| P07476 | IVL | 1.848579 | 0.001937 | 1.7765 | 0.7769 |
| P49023 | PXN | 1.819499 | 0.006446 | 1.92734 | 0.8538 |
| Q9NSB8 | HOMER2 | 1.818076 | 0.019845 | 1.50052 | 0.7077 |
| Q16644 | MAPKAPK3 | 1.811081 | 0.008423 | 1.69751 | 0.8038 |
| P55036 | PSMD4 | 1.801523 | 3.39E-05 | 2.12017 | 0.9192 |
| P56192 | MARS1 | 1.797437 | 0.012591 | 1.6742 | 0.8462 |
| Q15847 | ADIRF | 1.775476 | 0.016498 | 1.52733 | 0.7308 |
| Q8WVS4 | DYNC2I1 | 1.771884 | 0.005501 | 1.77943 | 0.7962 |
| Q5XUX1 | FBXW9 | 1.760552 | 0.004853 | 1.61394 | 0.7692 |
| O43768 | ENSA | 1.723282 | 0.001529 | 1.95068 | 0.8500 |
| Q8NFJ5 | GPRC5A | 1.719007 | 0.00533 | 1.55713 | 0.7769 |
| Q92783 | STAM | 1.691036 | 0.005619 | 1.59422 | 0.7885 |
| L0R6Q1 | SLC35A4 | 1.66454 | 0.014 | 1.69349 | 0.8308 |
| P84090 | ERH | 1.658783 | 0.001366 | 1.87871 | 0.8923 |
| O14907 | TAX1BP3 | 1.642973 | 0.016271 | 1.52633 | 0.7731 |
| P21127 | CDK11B | 1.642964 | 0.016788 | 1.63744 | 0.7154 |
| Q8TCA0 | LRRC20 | 1.638684 | 0.00052 | 2.04928 | 0.9077 |
| O60701 | UGDH | 1.637732 | 0.01446 | 1.52105 | 0.7346 |
| Q16674 | MIA | 1.636189 | 0.005694 | 1.58421 | 0.7423 |
| O60235 | TMPRSS11D | 1.627991 | 0.004672 | 1.66624 | 0.7962 |
| Q8IUE6 | H2AC21 | 1.622381 | 0.043065 | 1.6762 | 0.7423 |
| Q5TYW1 | ZNF658 | 1.619535 | 0.019246 | 1.58609 | 0.7654 |
| P0C0S8 | H2AC11 | 1.591844 | 0.006498 | 1.73161 | 0.7692 |
| Q9H1E3 | NUCKS1 | 1.589043 | 0.001431 | 2.00705 | 0.8500 |
| P02766 | TTR | 1.5795 | 0.002414 | 1.51376 | 0.7962 |
| Q06203 | PPAT | 1.579376 | 0.004583 | 1.71692 | 0.7962 |
| Q9UBG3 | CRNN | 1.571055 | 0.038117 | 1.50322 | 0.6615 |
| Q92890 | UFD1 | 1.568378 | 0.00521 | 1.69071 | 0.8038 |
| Q12802 | AKAP13 | 1.561112 | 0.004298 | 1.70583 | 0.7885 |
| Q96PC3 | AP1S3 | 1.54978 | 0.008586 | 1.64166 | 0.7731 |
| P34896 | SHMT1 | 1.541756 | 0.007623 | 1.6659 | 0.7808 |
| P06702 | S100A9 | 1.525737 | 0.012848 | 1.52718 | 0.7577 |
| Q3YEC7 | RABL6 | 1.517983 | 4.45E-05 | 1.98408 | 0.8692 |
| Q96B36 | AKT1S1 | 1.51791 | 0.001561 | 1.87031 | 0.8192 |
| Q9C0C2 | TNKS1BP1 | 1.516267 | 0.007369 | 1.8229 | 0.8269 |
| P62750 | RPL23A | 1.501226 | 0.001317 | 1.8215 | 0.8346 |
| P50897 | PPT1 | 0.664769 | 2.38E-05 | 2.178 | 0.9038 |
| Q13630 | GFUS | 0.664083 | 0.000446 | 1.97953 | 0.8538 |
| P52888 | THOP1 | 0.662529 | 0.000908 | 1.78595 | 0.8308 |
| P16435 | POR | 0.662286 | 2.32E-05 | 2.28167 | 0.8846 |
| P50281 | MMP14 | 0.658025 | 0.001681 | 1.81607 | 0.8346 |
| Q5SZK8 | FREM2 | 0.657054 | 0.000599 | 1.91562 | 0.8346 |
| Q9H8Y8 | GORASP2 | 0.649947 | 0.005924 | 1.76293 | 0.7962 |
| Q93088 | BHMT | 0.64324 | 0.002553 | 1.60386 | 0.7885 |
| P35754 | GLRX | 0.636681 | 0.003353 | 1.56003 | 0.8154 |
| Q0VDG4 | SCRN3 | 0.636315 | 0.001153 | 1.87037 | 0.8231 |
| O96019 | ACTL6A | 0.627873 | 0.000521 | 1.82739 | 0.8731 |
| Q9NPR2 | SEMA4B | 0.627748 | 0.016739 | 1.62904 | 0.7192 |
| Q9C002 | NMES1 | 0.627023 | 0.01255 | 1.67418 | 0.8269 |
| Q92538 | GBF1 | 0.624356 | 9.09E-08 | 2.54808 | 0.9615 |
| Q8IZ52 | CHPF | 0.62417 | 0.000511 | 1.97123 | 0.8500 |
| Q6KF10 | GDF6 | 0.621857 | 0.028037 | 1.68829 | 0.7308 |
| Q12899 | TRIM26 | 0.615682 | 0.001382 | 1.81041 | 0.8346 |
| Q9UKM7 | MAN1B1 | 0.615062 | 0.01069 | 1.72445 | 0.7692 |
| Q13131 | PRKAA1 | 0.60967 | 0.000413 | 1.94203 | 0.8808 |
| P22607 | FGFR3 | 0.603277 | 3.42E-05 | 2.21696 | 0.8885 |
| Q9Y2T3 | GDA | 0.602536 | 0.005002 | 1.79077 | 0.8154 |
| P49915 | GMPS | 0.600735 | 0.001953 | 1.57773 | 0.8269 |
| Q15582 | TGFBI | 0.589834 | 0.014522 | 1.79307 | 0.7308 |
| Q6UX06 | OLFM4 | 0.589476 | 0.005697 | 1.63166 | 0.7654 |
| Q16394 | EXT1 | 0.588728 | 0.002284 | 1.81957 | 0.8154 |
| Q9GZM7 | TINAGL1 | 0.584725 | 0.025681 | 1.59452 | 0.7154 |
| P14384 | CPM | 0.583863 | 0.000484 | 1.78106 | 0.8808 |
| Q9Y263 | PLAA | 0.576851 | 0.017808 | 1.59455 | 0.7615 |
| Q8WUJ3 | CEMIP | 0.572405 | 0.042815 | 1.58307 | 0.7077 |
| P08294 | SOD3 | 0.57155 | 0.017851 | 1.6168 | 0.7462 |
| P55291 | CDH15 | 0.561962 | 0.017202 | 1.74428 | 0.7808 |
| Q76KP1 | B4GALNT4 | 0.558196 | 0.017627 | 1.52341 | 0.7385 |
| Q15154 | PCM1 | 0.557315 | 0.032159 | 1.65104 | 0.7500 |
| P48960 | ADGRE5 | 0.553581 | 0.000312 | 1.85425 | 0.8654 |
| Q5EB52 | MEST | 0.553326 | 0.00089 | 1.99595 | 0.8269 |
| Q15274 | QPRT | 0.548305 | 0.011205 | 1.56467 | 0.7538 |
| Q9UJS0 | SLC25A13 | 0.532321 | 0.012897 | 1.53136 | 0.8077 |
| O60507 | TPST1 | 0.515955 | 1.66E-05 | 2.14663 | 0.8962 |
| Q01415 | GALK2 | 0.513557 | 0.017348 | 1.74953 | 0.7731 |
| P05997 | COL5A2 | 0.494082 | 0.027889 | 1.54363 | 0.7500 |
| P58215 | LOXL3 | 0.489393 | 0.004592 | 1.6223 | 0.7808 |
| Q8TD19 | NEK9 | 0.482685 | 0.006739 | 2.15564 | 0.8962 |
| P63000 | RAC1 | 0.479115 | 0.010273 | 2.08057 | 0.8423 |
| O75764 | TCEA3 | 0.47141 | 0.005193 | 1.68098 | 0.7885 |
| Q9NTJ4 | MAN2C1 | 0.462199 | 0.003907 | 2.10954 | 0.8423 |
| Q15111 | PLCL1 | 0.453655 | 0.017665 | 1.66707 | 0.7962 |
| Q9BZM4 | ULBP3 | 0.437432 | 1.66E-05 | 1.91794 | 0.9077 |
| Q15386 | UBE3C | 0.434112 | 0.005393 | 1.57827 | 0.8154 |
| Q8N436 | CPXM2 | 0.432975 | 4.44E-05 | 2.21271 | 0.9000 |
| P04085 | PDGFA | 0.422862 | 7.12E-11 | 2.86002 | 1.000 |
| O95456 | PSMG1 | 0.398872 | 4.55E-05 | 1.85625 | 0.8500 |
| Q9H2S5 | RNF39 | 0.383217 | 0.034958 | 1.57318 | 0.7115 |
| Q15147 | PLCB4 | 0.381493 | 0.008724 | 1.79746 | 0.7846 |
| Q07812 | BAX | 0.368393 | 3.37E-06 | 2.33919 | 0.9385 |
| P52294 | KPNA1 | 0.32281 | 1.6E-06 | 2.2151 | 0.9192 |
| P30519 | HMOX2 | 0.322737 | 2.79E-05 | 2.04654 | 0.8500 |
| P02778 | CXCL10 | 0.320788 | 0.008034 | 1.74958 | 0.7962 |
| A8K7I4 | CLCA1 | 0.314934 | 0.00394 | 1.55537 | 0.8423 |
| Q5SRE7 | PHYHD1 | 0.272415 | 0.028577 | 1.75576 | 0.7308 |
| P52333 | JAK3 | 0.252962 | 0.012721 | 1.77935 | 0.7577 |
| Q70IA6 | MOB2 | 0.186554 | 0.001171 | 2.28064 | 0.8192 |

**Table S3** Differentially expressed metabolites between the FGR-transient and FGR-persistent groups.

| Identity | VIP | *P*-Value | FC(FGR-persistent /FGR-transient) | AUC |
| --- | --- | --- | --- | --- |
| Pyrrolidine | 1.73084 | 0.007887 | 0.607122 | 0.8384 |
| 16alpha-Hydroxy DHEA 3-sulfate | 1.73116 | 0.020406 | 0.371145 | 0.7778 |
| N2-Succinoylarginine | 1.79005 | 0.02112 | 0.70397 | 0.7576 |
| Pentadecanedioylcarnitine | 1.64251 | 0.027804 | 0.537584 | 0.7273 |
| Leu-Trp | 1.70661 | 0.032187 | 0.524126 | 0.7576 |
| PC(16:1(9Z)/14:0) | 2.03872 | 0.032895 | 3.035058 | 0.7071 |
| cis-Aconitic acid | 1.80388 | 0.035292 | 0.655274 | 0.7576 |
| 2'-Deoxyuridine | 1.85892 | 0.035348 | 0.63961 | 0.7778 |
| 1-Methylguanine | 1.63596 | 0.043489 | 1.794553 | 0.7071 |
| 5-Amino-6-ribitylamino uracil | 1.60123 | 0.046552 | 0.475044 | 0.7182 |
| Pyroglutamic acid | 1.70541 | 0.047567 | 0.686484 | 0.7778 |
| 1-oleoyl-2-myristoyl-sn-glycero-3-phosphocholine | 1.9048 | 0.047597 | 2.114895 | 0.6869 |
| 7-Methylguanine | 1.63596 | 0.049403 | 1.706879 | 0.7071 |
| Arg-Ala | 1.62287 | 0.049894 | 0.52699 | 0.7677 |

**Table S4** Differentially expressed proteins between the FGR-transient and FGR-persistent groups.

| PG.Protein  Accessions | PG.Genes | VIP | *P* value | FC(FGR- transient  /FGR-persistent) | AUC |
| --- | --- | --- | --- | --- | --- |
| P10321 | HLA-C | 1.97505 | 0.00023 | 3.868479 | 0.9495 |
| Q9UMS0 | NFU1 | 2.66522 | 0.000291 | 0.342178 | 0.9192 |
| P48200 | IREB2 | 2.81146 | 0.000316 | 0.245081 | 0.9697 |
| Q9Y4E1 | WASHC2C | 2.08759 | 0.000481 | 2.517334 | 0.9192 |
| Q8IYJ2 | C10orf67 | 1.94428 | 0.000996 | 3.906897 | 0.8889 |
| Q13296 | SCGB2A2 | 2.09956 | 0.001 | 2.055567 | 0.9192 |
| Q9UKY7 | CDV3 | 1.65781 | 0.001233 | 2.337092 | 0.8687 |
| O15031 | PLXNB2 | 1.77723 | 0.00217 | 2.662827 | 0.9394 |
| Q9NUJ3 | TCP11L1 | 2.15142 | 0.0022 | 2.154361 | 0.8687 |
| Q9GZM5 | YIPF3 | 1.09968 | 0.003056 | 0.116316 | 0.8485 |
| Q13042 | CDC16 | 1.87871 | 0.003483 | 0.368723 | 0.8788 |
| Q9UK85 | DKKL1 | 1.97104 | 0.003674 | 0.380494 | 0.8485 |
| Q9P2K3 | RCOR3 | 2.53791 | 0.003868 | 0.331891 | 0.8788 |
| P41222 | PTGDS | 1.31597 | 0.003875 | 0.48069 | 0.8889 |
| Q70IA6 | MOB2 | 1.55097 | 0.004474 | 0.329877 | 0.8485 |
| Q9H9C1 | VIPAS39 | 2.14415 | 0.005699 | 0.454153 | 0.8384 |
| P51911 | CNN1 | 1.98268 | 0.006506 | 2.508463 | 0.8586 |
| O00300 | TNFRSF11B | 1.39015 | 0.006929 | 2.551413 | 0.8889 |
| P80217 | IFI35 | 1.59527 | 0.007878 | 0.349907 | 0.8283 |
| Q9BZL1 | UBL5 | 2.09148 | 0.007917 | 0.4463 | 0.8687 |
| P17031 | ZNF26 | 1.58614 | 0.008 | 3.931054 | 0.7677 |
| Q9P0K1 | ADAM22 | 1.58138 | 0.00846 | 0.370058 | 0.8485 |
| P14317 | HCLS1 | 1.69555 | 0.008752 | 2.360857 | 0.8182 |
| Q9UJF2 | RASAL2 | 1.62995 | 0.009205 | 0.416223 | 0.8384 |
| Q9BXU1 | STK31 | 1.25634 | 0.009315 | 0.410093 | 0.8081 |
| Q92990 | GLMN | 1.83498 | 0.009325 | 0.417337 | 0.8182 |
| Q9ULT0 | TTC7A | 1.5312 | 0.009433 | 2.669105 | 0.8182 |
| P08473 | MME | 1.63391 | 0.009952 | 0.381489 | 0.8283 |
| P12273 | PIP | 1.81587 | 0.01119 | 2.017585 | 0.8384 |
| Q9HAV0 | GNB4 | 2.04052 | 0.01127 | 2.264576 | 0.8485 |
| O00217 | NDUFS8 | 2.10988 | 0.011761 | 0.456531 | 0.8384 |
| Q96T76 | MMS19 | 1.6822 | 0.012196 | 0.437148 | 0.8081 |
| Q9BZM4 | ULBP3 | 1.73765 | 0.01242 | 0.493524 | 0.8081 |
| Q9H479 | FN3K | 1.50676 | 0.013592 | 0.307555 | 0.8081 |
| Q5VSL9 | STRIP1 | 1.68559 | 0.015154 | 0.222319 | 0.8283 |
| Q13287 | NMI | 2.01479 | 0.016672 | 0.479431 | 0.7879 |
| P02741 | CRP | 0.982992 | 0.019054 | 2.557605 | 0.8182 |
| P22455 | FGFR4 | 1.70189 | 0.019149 | 0.427088 | 0.8384 |
| Q7Z404 | TMC4 | 1.51777 | 0.019805 | 2.33715 | 0.7778 |
| Q9GZX9 | TWSG1 | 1.14869 | 0.019995 | 0.465294 | 0.7980 |
| O76061 | STC2 | 1.88972 | 0.020448 | 2.002979 | 0.8081 |
| O00533 | CHL1 | 1.60274 | 0.02081 | 2.080844 | 0.7576 |
| Q9Y3Y2 | CHTOP | 1.72747 | 0.021275 | 2.669939 | 0.7374 |
| Q9Y587 | AP4S1 | 1.63183 | 0.021308 | 0.328078 | 0.8182 |
| Q16637 | SMN1 | 1.83594 | 0.022271 | 0.493817 | 0.8182 |
| P05413 | FABP3 | 1.46983 | 0.023354 | 5.293047 | 0.8586 |
| Q13162 | PRDX4 | 1.86009 | 0.023468 | 2.048062 | 0.7677 |
| P00918 | CA2 | 1.84151 | 0.023624 | 2.005823 | 0.8081 |
| P53384 | NUBP1 | 1.82669 | 0.02365 | 0.409591 | 0.7879 |
| Q9NZT1 | CALML5 | 1.27549 | 0.024012 | 2.019625 | 0.8384 |
| P51888 | PRELP | 1.52 | 0.025364 | 7.192158 | 0.8182 |
| Q9NTJ4 | MAN2C1 | 1.69941 | 0.026353 | 0.484187 | 0.8081 |
| P20929 | NEB | 1.68583 | 0.026443 | 0.467078 | 0.8081 |
| O60238 | BNIP3L | 1.76646 | 0.027804 | 0.461157 | 0.7879 |
| Q6ZW49 | PAXIP1 | 1.77535 | 0.027823 | 0.26971 | 0.7879 |
| Q17R89 | ARHGAP44 | 1.56149 | 0.029582 | 0.190972 | 0.7778 |
| A0A0B4J2D9 | IGKV1D-13 | 1.26562 | 0.031898 | 2.024346 | 0.7677 |
| Q9UMD9 | COL17A1 | 1.3764 | 0.033852 | 3.813767 | 0.8283 |
| P23634 | ATP2B4 | 1.41401 | 0.037006 | 2.125981 | 0.7273 |
| Q16352 | INA | 1.87633 | 0.039338 | 2.311333 | 0.7980 |
| Q86XP0 | PLA2G4D | 1.51194 | 0.041676 | 0.466624 | 0.7576 |
| O95373 | IPO7 | 1.51979 | 0.043758 | 0.497676 | 0.8283 |
| Q8WYN0 | ATG4A | 1.4426 | 0.043964 | 0.149255 | 0.7576 |
| P11441 | UBL4A | 1.28041 | 0.044527 | 0.495461 | 0.7273 |
| P50591 | TNFSF10 | 1.0288 | 0.04476 | 2.21987 | 0.7071 |
